# Portdysfunktion und bronchopulmonale Symptomatik

**DOI:** 10.1007/s00117-025-01548-5

**Published:** 2026-01-06

**Authors:** Christopher Kloth, Aida Eren, Gunter Lang, Adela-Maria Neagoie, Jochen Klaus, Stefan Andreas Schmidt, Meinrad Beer, Daniel Vogele

**Affiliations:** 1 Klinik für diagnostische und interventionelle Radiologie, Universitätsklinikum Ulm, Albert-Einstein-Allee 23, 89081 Ulm, Deutschland; 2 Praxis für Radiologie und Strahlentherapie Lindau, Friedrichshafener Str. 82/83, 88131 Lindau/Bodensee, Deutschland; 3 Klinik für Herz‑, Thorax- und Gefäßchirurgie, Universitätsklinikum Ulm, Ulm, Deutschland; 4 Klinik für Innere Medizin III, Universitätsklinikum Ulm, Ulm, Deutschland; 5 Klinik für Innere Medizin II, Universitätsklinikum Ulm, Ulm, Deutschland

## Anamnese

Wir berichten über einen 23-jährigen Patienten, der sich mit bronchopulmonaler Symptomatik in unserer Klinik vorstellte. Als Hauptdiagnose war ein Morbus Crohn mit chronisch aktivem Verlauf bekannt. In der Vorgeschichte erfolgten bereits mehrfache Ileumteilresektionen bei Perforation mit Abszess und teils enterokutaner Fistelbildung. Die aktuelle Therapie erfolgte mit Azathioprin und Infliximab alle 4 Wochen. Zur medikamentösen Behandlung und heimparenteralen Ernährung aufgrund eines Malabsorptionssyndroms bei Verdacht auf ein funktionelles Kurzdarmsyndrom wurde ein Portkatheter über die linke Vena cephalica implantiert. Die Implantation des Portkatheters erfolgte bereits 19 Monate vor der hier berichteten Vorstellung in der Klinik. Der Patient berichtete über einen seit 2 Tagen bestehenden Hustenreiz. Zudem klagte er über Geschmacksmissempfindungen nach der parenteralen Ernährung über den Portkatheter. Fieber, Auswurf oder andere Erkältungssymptome wurden verneint. Bei der Laboruntersuchung zeigte sich das C‑reaktive Protein (CRP) mit 39,1 mg/l (Referenzbereich < 5,0 mg/l) erhöht bei normwertiger Leukozytenzahl.

## Radiologische Diagnostik

**Abb. 1:**
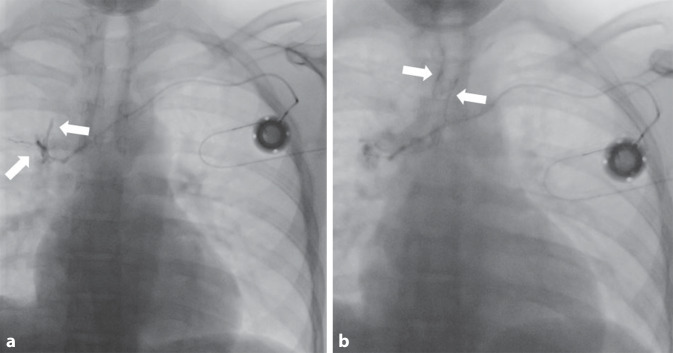
Durchleuchtungsuntersuchung des Thorax mit Kontrastmittelgabe über den Portkatheter. Es zeigt sich zunächst eine Kontrastierung des Bronchialsystems (*Pfeile*) im rechten Oberlappen (**a**) und in der Folge auch der Trachea (*Pfeile*) bei Hustenreiz des Patienten (**b**)

Zur weiteren Diagnostik wurde eine Durchleuchtungsuntersuchung des Portkatheters durchgeführt. In der initialen Leeraufnahme projizierte sich die Spitze des Portkatheters auf die Vena cava superior. Das Anspülen mit NaCl-Lösung war problemlos möglich, hierbei jedoch direkter Hustenreiz des Patienten auslösbar. In der anschließend angefertigten Durchleuchtungsserie unter Kontrastmittelapplikation zeigte sich eine Kontrastierung des an die Portspitze angrenzenden Bronchialsystems (Abb. 1a). Auch hierunter war ein deutlicher Hustenreiz auslösbar mit Nachweis von Kontrastmittel in der Trachea (Abb. 1b).

**Abb. 2:**
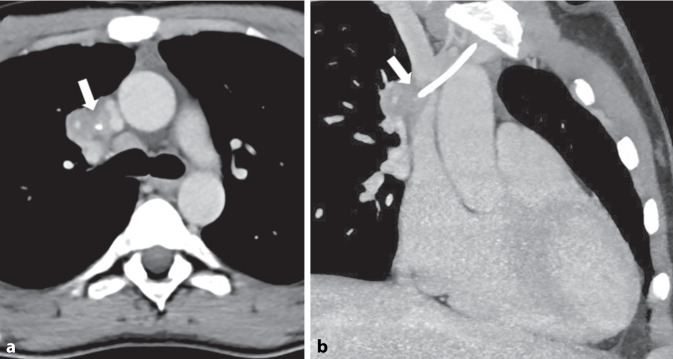
Computertomographie (*CT*) des Thorax mit axialer (**a**) und parakoronarer (**b**) Rekonstruktion im Weichteilfenster. Um die Spitze des Portkatheterschlauchs ist ein Thrombus abgrenzbar (*Pfeile*) mit angrenzendem Hämatom. Die Spitze des Portkatheters scheint neben der Vena cava superior zu liegen

In der ergänzenden kontrastmittelgestützten Computertomographie (CT) zeigte sich ein Thrombus in der Vena cava superior (VCS) um die Portkatheterspitze. Die Portkatheterspitze schien knapp außerhalb der VCS zu liegen mit umgebendem Hämatom (Abb. 2).

## Wie lautet Ihre Diagnose?

## Definition

Zentralvenöse Portkatheter sind für Patienten indiziert, die eine langfristige intravenöse Therapie benötigen [1]. Dies umfasst onkologische Patienten mit der Notwendigkeit einer Chemotherapie, Patienten mit parenteraler Ernährung oder regelmäßigen Infusionen ebenso wie Patienten, bei denen aufgrund von Gefäßanomalien oder Gefäßverschlüssen ein sonstiger Zugangsweg nicht möglich ist. Alternativen sind nicht vollständig implantierbare zentralvenöse Katheter wie ein peripher eingeführter zentralvenöser Katheter, auch PICC-Line genannt („peripherally inserted central venous catheter“ [2]).

Als ideale Position der distalen Katheterspitze wird die distale obere Hohlvene (VCS) angesehen. Das große Blutvolumen in einer großkalibrigen Vene verdünnt die verabreichte Medikation sofort und verringert das Risiko von Gefäßschäden. Dies ist besonders wichtig bei Chemotherapeutika, die in Lösungen mit hoher Osmolalität verabreicht werden [1]. Es ist bekannt, dass diese die Gefäßwand schädigen können mit möglichen Folgekomplikationen wie Infektionen und thrombotischem Verschluss oder Gefäßstenosierung [1].

Allgemein kann man Frühkomplikationen (< 30 Tage nach Implantation) gegenüber Spätkomplikationen (> 30 Tage nach Implantation) unterscheiden [1]. Eine Übersicht zeigt Tab. 1.

**Tab. 1:** Übersicht über die häufigsten Früh- und Spätkomplikationen bei Portkatheterimplantationen. (Adaptiert aus [1])

Frühkomplikationen	Spätkomplikationen
Fehlpositionierung	Spitzenthrombose
Pneumothorax	Gefäßthrombose
Hämatothorax	Katheterbruch
Infektion	Infektion
Chylothorax/Verletzung Ductus thoracicus	

Die Rate an Komplikationen variiert stark zwischen 0,3 und 12,5 %, hierbei führt die Komplikation der Portkatheterinfektion sowie der Gefäßthrombose [1, 3, 4].

Bildgebend können verschiedene Modalitäten zur Beurteilung eines Portsystems herangezogen werden. Zum Ausschluss direkter Komplikationen nach Implantation – wie einer Fehllage – sollte eine ergänzende Röntgenaufnahme des Thorax erfolgen. Primär sollte die Gefäßbeurteilung mittels Sonographie erfolgen. Der Sonographie ist zumeist die Vena subclavia, die Vena jugularis oder Vena axillaris zugänglich, um eine etwaige assoziierte Gefäßthrombose im proximalen Verlauf des Portschlauchs zu eruieren. Zur Beurteilung des zentralen Gefäßverlaufes hingegen ist in der Regel eine kontrastangehobene CT in venöser Phase zielführend. Die Portkatheterspitze ist in Abhängigkeit der Herzbewegung unterschiedlich gut bewertbar. Die Applikation des Kontrastmittels von kontralateral kann dabei helfen, Aufhärtungsartefakte zu vermeiden. Die Durchleuchtungsmethode bietet die Möglichkeit der Darstellung auch kleiner Katheterspitzen-assoziierten Thromben sowie die Darstellung etwaiger gefäßassoziierter Komplikationen wie der arteriovenösen Fistel. Die Komplikation der venotrachealen Fistel bzw. der venobronchialen Fistel wie im vorliegenden Fall ist in der Literatur nur in Form von Einzelfallberichten beschrieben [4–6]. Zumeist handelt es sich um eine sekundäre Dislokation der Portkatheterspitze als Spätkomplikation bzw. um ein mechanisches Herausarbeiten der Katheterspitze durch Arrosion der Wand der VCS. Die zugrundeliegende Literatur diskutiert hierbei als prädisponierende Faktoren die Gabe aggressiver Chemotherapeutika sowie eine enge anatomische Nähe der VCS zum rechten Hilus [5].

**Diagnose: **Portkatheter mit venobronchialer Fistel

Zu bedenken gilt es dabei, dass die Hauptdiagnose des Patienten im vorliegenden Fall ein chronisch aktiver Morbus Crohn ist. Im Rahmen chronisch-entzündlicher Darmerkrankungen sind Beteiligungen der Lunge in Form interstitieller Lungenerkrankungen, Erkrankungen des Tracheobronchialsystems (Bronchiektasen, Bronchiolitis) sowie auch der thorakalen Gefäße (Vaskulitis) möglich. So finden sich auch Beschreibungen bronchopulmonaler Fisteln [7]. Die klinische Symptomatik variiert von Nacken‑/Schulterschmerzen bis hin zu bronchopulmonaler Symptomatik [4]. Auch die applizierten Medikamente Infliximab und Azathioprin können als Nebenwirkung unspezifische bronchopulmonale Symptome verursachen.

Im vorliegenden Fall war die führende Symptomatik ein neu aufgetretener Hustenreiz und Geschmacksmissempfindungen in engem zeitlichem Zusammenhang mit der Gabe der parenteralen Ernährung über den Portkatheter. Die Implantation des Katheters lag bereits 19 Monate zurück. Die rasche Initiierung bildgebender Diagnostik ist in solchen Fällen wichtig, um potenziell schwerwiegende Komplikationen frühzeitig zu diagnostizieren.

Zusammenfassend ist die venobronchiale Fistel als äußerst seltene Komplikation zu benennen. Wichtig ist es, diese zu kennen und insbesondere bei repetitivem Husten im Zusammenhang mit der Nutzung des Portkatheters zu bedenken.

## Therapie und Verlauf

Zunächst wurde ein interventionell-radiologisches Vorgehen diskutiert. Aufgrund der bestehenden Fistel und einer potenziellen Notwendigkeit der operativen Cava-Revision wurde in der interdisziplinären Diskussion ein chirurgisches Vorgehen favorisiert. Intraoperativ wurde der Portschlauch zunächst vorsichtig zurückgezogen und Kontrastmittel appliziert. Hierbei bestätigte sich der Befund der venobronchialen Fistel. Nach weiterem Rückzug kontrastierte sich ein Kanal wie bei einliegendem Katheter, der in Richtung der Fistel reichte, sodass von einem organisierten Thrombus um den Portschlauch auszugehen war. Nach Punktion über die Gegenseite war die Fistel mittels einer durchgeführten Cavographie nicht nachweisbar, sodass der linksseitige Portkatheter vollständig entfernt wurde. Über die rechte Seite konnte anschließend komplikationslos ein neues Portkathetersystem implantiert werden. Aufgrund des Thrombus wurde eine orale Antikoagulation mit Rivaroxaban 20 mg für 3 Monate verabreicht. Der neu implantierte Portkatheter konnte in der Folge komplikationslos verwendet werden.

## Fazit für die Praxis

- Die venobronchiale Fistel ist eine äußerst seltene Komplikation nach Implantation eines Portkatheters.
- Die venobronchiale Fistel kann mit langem zeitlichem Abstand nach der Portimplantation auftreten.
- An eine venobronchiale Fistel ist bei bronchopulmonaler Symptomatik und Geschmacksmissempfindungen im Zusammenhang mit der Nutzung eines Portkatheters zu denken.
- Die zugrundeliegende chronisch-entzündliche Darmerkrankung wie auch die Gabe potenziell gefäßschädigender Medikamente können dabei als begünstigende Faktoren angesehen werden.
